# Dietary supplementation with plant extracts for amelioration of persistent myofascial discomfort in the cervical and back regions: a randomized double-blind controlled study

**DOI:** 10.3389/fnut.2024.1403108

**Published:** 2024-06-03

**Authors:** Silvia Pérez-Piñero, Juan Carlos Muñoz-Carrillo, Jon Echepare-Taberna, Antonio J. Luque-Rubia, Jose Eduardo Millán Rivero, Macarena Muñoz-Cámara, María Josefa Díaz Silvente, Eloina Valero Merlos, Vicente Ávila-Gandía, Nuria Caturla, Pau Navarro, María Cabrera, Francisco Javier López-Román

**Affiliations:** ^1^Faculty of Medicine, UCAM Universidad Católica San Antonio de Murcia, Murcia, Spain; ^2^Health Sciences PhD Program, UCAM Universidad Católica San Antonio de Murcia, Murcia, Spain; ^3^Faculty of Nursing, UCAM Universidad Católica San Antonio de Murcia, Murcia, Spain; ^4^Monteloeder S.L., Elche, Spain; ^5^Primary Care Research Group, Biomedical Research Institute of Murcia (IMIB-Arrixaca), Murcia, Spain

**Keywords:** back pain, rosemary, ashwagandha, sesame, quality of life, herbal extracts, randomized clinical trial, food supplement

## Abstract

**Background:**

Back pain is a common health problem that affects both workers and older people, reducing their quality of life. The primary objective was to assess the effect of dietary supplementation with plant extracts of rosemary, ashwagandha, and sesame consumed for 12 weeks on the intensity of back pain.

**Methods:**

A single-center randomized double-blind study with three parallel arms depending on the product consumed. The duration of treatment was 12 weeks. The investigational product, Berelief^®^, contained a blend of three polyphenolic standardized extracts: rosemary (*Rosmarinus officinalis* L.), ashwagandha (*Withania somnifera* L.), and sesame (*Sesamum indicum* L.) seed. Two doses were tested: low dose (400 mg) and high dose (800 mg). There were 42 subjects in the placebo group, 39 in the low dose and 42 in the high dose groups. Study variables included back pain intensity [VAS score, Patient-Reported Outcomes Measurement Information System (PROMIS-29), and Cornell Musculoskeletal Discomfort Questionnaire; functionality Roland-Morris Disability (RMD) questionnaire]; quality of life (QoL) [36-item Short Form Survey (SF-36), the Beck Depression Inventory-II (BDI-II), the State–Trait Anxiety Inventory (STAI), and the Perceived Stress Scale (PSS)]; sleep quality [accelerometer and Pittsburgh Sleep Quality Index (PSQI)].

**Results:**

The improvement in back pain recorded by the visual analogue scale (VAS) at the study visits after the beginning of treatment, as well as on a weekly basis recorded in the diary card was significantly higher in the intervention group than in the placebo group (*p* < 0.044 dose-low; *p* < 0.005 dose-high). Significant differences in pain intensity of the PROMIS-29 (*p* = 0.002) and upper back pain in the Cornell questionnaire (*p* = 0.011) in favour of the investigational product were found. Furthermore, benefits in improving health-related quality of life, mood and sleep quality were also detected.

**Conclusion:**

Dietary supplementation for 12 weeks of a blend of polyphenolic standardized extracts of rosemary, ashwagandha, and sesame was effective in reducing the intensity of pain in subjects with chronic myofascial cervical and back pain.

## 1 Introduction

In today’s hectic lifestyle, many people experience some form of discomfort that negatively affects their ability to carry out daily activities efficiently. The escalating prevalence of discomfort experienced by individuals is primarily attributed to the on-going demographic change and concomitant population aging, as well as unhealthy lifestyles. If these episodic aches are not managed properly, they can turn into chronic conditions and led to more severe problems.

Back pain, whether in the upper back or lumbar area, is one of the most frequent sources of pain due to musculoskeletal disorders and is an important public health problem that can affect quality of life (1). Also, it is one of the most common reasons for adult patients to seek medical care both in the primary and emergency care setting (2) and represents the highest percentage of referrals and workload for physical therapy utilization (3). Back pain is widespread in the adult population leading to great economic and social costs (4). Estimates from the Global Burden of Disease Study 2017 have shown an age-standardized point prevalence for lower back pain of 8.2%, higher in females than males, and increasing with age (5). Systematic reviews have provided evidence of a high prevalence of chronic lower back pain in older adults, with a variable pooled 12-month prevalence of 21 to 68% (6, 7). Since the world population of adults aged 60 years or older is estimated to increase from 1 billion in 2020 to 1.4 billion in 2030 (with 2.1 billion in 2050) (8), it is critically important to identify proper prevention and treatment strategies to be implemented for individuals at risk.

In most people, acute back pain symptoms resolve spontaneously, but in some patients the symptoms continue and become persistent or even chronic (longer than 12 weeks despite treatment). Clinical studies have searched for signs or parameters for predicting pain chronicity, but no consistent neurobiological, behavioural or psychological factors have emerged (9, 10). Chronic back pain management depends on whether the pain is specific (can be explained by an underlying cause) or non-specific/idiopathic, being the latter case the most common (11). Although there are numerous pharmacological and non-pharmacological treatments, including pain-killers, physical therapies, lifestyle changes, education, self-care, and psychological support, clinical trials evaluating the efficacy of a variety of treatment indicate limited efficacy for the majority of the commonly applied interventions and approaches (12). A systematic review from the American College of Physician Practice Guideline of pharmacological therapies for lower back pain concluded that systemic medications [acetaminophen, non-steroidal anti-inflammatory drugs (NSAIDs), skeletal muscle relaxants, benzodiazepines, systemic corticosteroids, anti-seizure medications, and opioids] were ineffective or associated with small to moderate primarily short-term effects (13). On the other hand, non-pharmacological measures, such as exercise, multi-disciplinary rehabilitation, psychological therapies, spinal manipulation, massage therapy, mindfulness-based stress reduction, or acupuncture have shown to provide small short-term benefits (14).

Due to the limited effectiveness, increased risks, or adverse effects of available medication options, there is a need for safe therapeutic alternatives that can reduce pain and complement other treatment methods. Adopting healthy habits such as regular exercise, balanced nutrition, and maintaining a healthy weight can be effective in preventing chronic pain. However, in some cases, these habits may not be sufficient. In such cases, food supplements can help alleviate pain and enable people to engage in other active treatment modalities as part of a holistic and comprehensive treatment plan.

Although nutritional supplements are growing in popularity, there is limited scientific evidence regarding their efficacy. Rigorous scientific inquiry is needed to determine their true efficacy and safety. Botanical products have recently gained increasing attention for their potential to reduce pain and improve function in chronic non-specific lower back pain. However, evidence of the benefits of herbal medicine in the back pain setting is scarce. A systematic review of 14 randomized controlled studies with 2050 patients with acute, subacute, or chronic non-specific low back pain found some improvements in pain and functional status during 4 to 6 weeks with the use of *Solidago chilensis* (Brazilian arnica) gel, *Capsicum frutescens* cream, and oral doses of *Harpagophytum procumbens* (devil’s claw) (standardized 50 or 100 mg harpagoside), *Salix alba* (white willow bark), (standardized to 120 mg or 240 mg salicin), and *Symphytum officinale* L. (comfrey root extract) compared to placebo (15). In another in-depth review of the management of chronic low back pain with herbal, vitamin, mineral, and homeopathic supplements, the use of *Camphora molmol*, *Capsicum frutescens*, *Salix alba*, *Maleluca alternifolia*, *Angelica sinensis*, *Aloe vera*, *Thymus officinalis*, *Menthe peperita*, *Arnica montana*, *Curcuma longa*, *Tancaetum parthenium*, *Harpagophytum procumbens*, and *Zingiber officinalis* showed some short-term beneficial effect versus placebo (16). It should be noted that the efficacy of a combination of herbal extracts has not been evaluated in none of these studies. It is possible that supplementation with a mixture of herbal products may produce a synergistic effect, potentially increasing the effect of each compound and further reducing back pain symptoms.

For this reason, it was considered of interest to design a randomized controlled clinical trial to assess the benefits of a herbal ingredient composed of rosemary, ashwagandha, and sesame seed extracts, administered in two different doses, in subjects with chronic myofascial back pain compared to placebo.

## 2 Materials and methods

### 2.1 Study design

A single-center randomized double-blind study with three parallel arms depending on the product consumed (investigational product dose 1 or low dose and dose 2 or high dose, and placebo) was conducted at the Department of Health Sciences of San Antonio Catholic University of Murcia (UCAM), in Murcia, Spain. The study period was from September 21, 2022 to April 26, 2023.

The study was conducted in accordance with the World Medical Association’s (WMA) Helsinki Declaration and its amendments. Both the study protocol and the in-formed consent form were approved by the Ethics Committee of San Antonio Catholic University (code CE062205 approval date July 24, 2022) (Murcia, Spain) and was registered in the ClinicalTrials.gov (NCT05597189).

The primary objective was to assess the effect of the study products consumed for 12 weeks on the intensity of back pain. Secondary objectives included reduction in the frequency and/or amount of analgesic medication; assessment of the degree of back pain and functionality using patient-centered outcome measures; health-related quality of life; sleep quality; inflammatory biomarkers; body composition; level of physical activity; and safety.

### 2.2 Eligibility criteria for participants

Participants were recruited from the database for clinical studies available at UCAM. Inclusion criteria included healthy men and women aged between 20 and 65 years, episodic persistent myofascial back pain (cervical, dorsal, lumbar areas) with a value of at least 3 using a 1–10 cm visual analogue scale (VAS) and for at least 3 months, and body mass index (BMI) between 18.5 and 29.9 kg/m^2^. Exclusion criteria were as follows: injury-associated pain; pain caused by chronic conditions, such as rheumatoid arthritis, herniated disks, ankylosing spondylitis, etc.; severe or terminal illnesses; subjects with known allergy to any of the components of the investigational product; subjects undergoing physiotherapy treatment during the course of the study; pregnant or lactating women; and inability to understand the informed consent.

In addition, subjects are requested to respect the following requirements during the whole study (after inclusion): avoid initiating or altering hormonal/medical treatments without justification; abstain from treatments affecting study parameters; refrain from consuming food supplements and avoid modifying regular dietary patterns, particularly in relation to flavonoid-rich foods such as fruits, vegetables, coffee, etc.; and maintain consistent physical activity habits throughout the study. A signed informed consent form was obtained from all the subjects participating in the study before any study-related procedure took place.

### 2.3 Randomization, masking and study groups

Eligible subjects who provided the written informed consent were randomized to one of the three study groups (1:1:1) using a computer-generated randomization list with the Epidat 4.1 software program. The study groups were as follows: (a) low dose of the investigational product, (b) high dose of the investigational product, and (c) placebo. Both investigators and participants remained blinded to group assignments. Products were coded with unique numbers, detailed on a sheet indicating subject and product codes. The randomization sheet, signed and dated, revealed the product assignments only at the study’s conclusion.

The investigational product was a commercially available food supplement ingredient (Berelief^®^) supplied by Monteloeder, S.L. (Elche, Alicante, Spain), which is a botanical blend of three standardized herbal extract. Specifically, it contains 66% of ashwagandha (*Withania somnifera* L.) root extract standardized in whitanolides by high-performance liquid chromatography (HPLC), 22% sesame (*Sesamum indicum* L.) seed extract standardized in sesamin by HPLC, and *Rosmarinus officinalis* extract standardized in carnosic acid by HPLC. In total, w/w, this blend comprises a minimum content of 2.5% carnosic acid, 2.8% withanolides, and 5.5% sesamin. The ingredients present in the final formula included transient receptor potential vanilloid 1 (TRPV1), the voltage-gated sodium channel isoform 1.7 (NaV1.7), and tropomyosin receptor kinase A (TrkA), which were selected for their individual and complementary effects in inhibiting ion channels involved in pain-related signaling cascades (data not shown).

In the case of low dose, in each jelly capsule 36.4% (200 mg) of the product was the botanical blend ingredient, 45.4% microcrystalline cellulose, and 18.2% encapsulation, whereas in high dose, capsules were composed of 66.7% botanical blend ingredient (400 mg), 16.7% microcrystalline cellulose, and 16.6% encapsulation. The placebo product was composed of 18.2% magnesium stearate, 63.6% microcrystalline cellulose, and 18.2% of the capsule. Both the dietary supplement and placebo products were in opaque colored capsules with identical appearances. They were pre-packed in blisters and consecutively numbered for each subject according to the randomization list.

Subjects were instructed to take two capsules of the assigned product, 200 and 400 mg per capsule in the low and high dose groups, respectively, or placebo capsules, once a day, 30 min before breakfast for 84 consecutive days (12 weeks).

### 2.4 Study procedures and compliance

The study included a screening visit (visit 0), a baseline visit (visit 1) and two intermediate visits at 4 (visit 2) and 8 (visit 3, end of study) weeks followed by a final visit at week 12 (visit 4). The screening visit took place within ±7 days prior to the baseline visit, in which the inclusion criteria were checked, the written informed con-sent was obtained, and randomization was performed.

At the baseline visit (visit 1), subjects received the study product, a diary, and a sleep accelerometer and the following variables were recorded: concomitant medication; back pain intensity [VAS score, Patient-Reported Outcomes Measurement Information System (PROMIS-29)], and Cornell Musculoskeletal Discomfort Questionnaire; functionality [Roland-Morris Disability (RMD) questionnaire]; quality of life (QoL) [36-item Short Form Survey (SF-36), the Beck Depression Inventory-II (BDI-II), the State–Trait Anxiety Inventory (STAI), and the Perceived Stress Scale (PSS)]; sleep quality [accelerometer and Pittsburgh Sleep Quality Index (PSQI)].

Concomitant medication, study variables recorded at visit 1 (except for subjective sleep quality and physical activity), and adverse events were evaluated at visits 2 and 3. At the final visit (visit 4), all study variables were assessed, the study product and the diary were collected, adverse events were registered, and a blood sample was drawn for analyses of inflammatory markers and safety testing.

Compliance at the final visit was defined as the number of capsules taken by the participant during the study, divided by the number of capsules expected to be taken (*n* = 168), and multiplied by 100. Subjects were required to consume at least 80% of the total treatment, so they could only leave 34 capsules unconsumed corresponding to 17 out of the 84 days of supplementation.

### 2.5 Study variables

Clinical variables included age, sex, body mass index (BMI), blood pressure, and percentage of decrease of concomitant analgesic medication.

Pain was the primary efficacy variable and was measured using a 1–10 cm VAS scale (0 = no pain, 10 = worst pain imaginable) and the PROMIS-29 and the Cornell questionnaires. VAS scores were assessed at the study visits and also recorded daily by the study subjects on their diary cards.

PROMIS-29 (v2.0) is a 29-item validated questionnaire (17, 18) that assesses 7 do-mains: pain interference, physical function, anxiety, depression, fatigue, sleep disturbance, satisfaction with participation in social roles, and finally a pain intensity scale. The first seven domains are assessed with 4 questions each. On symptom-oriented (negatively worded) domains of PROMIS-29 (anxiety, depression, fatigue, pain interference, and sleep disturbance), higher scores represent worse symptomatology. On the function-oriented (positively worded) domains (physical function and social role) higher scores represent better functioning.

The Cornell Musculoskeletal Discomfort Questionnaire is a 54-item questionnaire developed for sedentary and standing workers (19) that includes a body chart and questions about occurrence of pain (frequency, intensity, and work interference) in 20 parts of the body over the last work week. Firstly, the level of discomfort recorded by the subject is calculated as “never (0), 1 or 2 times/week (1.5), 3 or 4 times/week (3.5), every day (5), or several times every day (10).” In order to reach the weighted musculoskeletal discomfort level, the result is then multiplied by the severity rate (“slightly uncomfortable = 1, moderately uncomfortable = 2, very uncomfortable = 3”) and interference rating (“not at all = 1, slightly interfered = 2, substantially interfered = 3”). Thus, the product of the weighted responses on the three scales gives a weighted score for each body part which ranges between 0 (i.e., “never” on the frequency scale) and 90 (i.e., 10 on the frequency scale x 3 on the severity scale x 3 on the work interference scale). The following areas were independently evaluated: neck, shoulder (right, left), upper back, and lower back.

The Roland Morris Disability (RMD) questionnaire is a 24-item patient-reported outcome measure inquiring about pain-related disability resulting from low back pain. Items are scored 0 if left blank or 1 if endorsed, for a total RMD score ranging from 0 to 24; higher scores represent higher levels of pain-related disability. A change of ≥2 points is clinically relevant (20). A Spanish validated version was used (21).

The SF-36 is a 36-item scale which measures eight domains of health status: physical functioning, physical role limitations, bodily pain, general health perceptions, energy/vitality, social functioning, emotional role limitations, and mental health. A health transition question (HTQ) estimates changes in health status compared to the previous year. Scores of the eight dimensions are transformed to range from 0 where the respondent has the worst possible health to 100 where the respondent is in the best possible health. A Spanish validated version of the instrument was used (22, 23).

The BDI-II is a 21-item rating questionnaire to assess symptoms of depression that occurred during the previous month. Each question has four possible answers, scoring from 0 to 3. The total score ranges from 0 to 63. In non-clinical populations, scores above 20 indicate depression. In those diagnosed with depression the standard cutoff values are 0–13 for minimal depression, 14–19 for mild, 20–28 for moderate, and 29–63 for severe. A Spanish validated version was used (24).

The STAI questionnaire measures state (STAI-state) and trait (STAI-trait) of anxiety based on 20 questions for each domain, scores can vary between 0 and 60 with higher scores indicating greater anxiety levels. A Spanish validated version was used (25).

The PSS scale includes 14 items measuring the frequency or extent of a certain stress-signaling event occurrence of a 5-point scale (from 0: never to 4: very often). Total perceived stress level score ranges between 0 and 56 (scores 0–18 indicate low stress, scores 19–37 indicate moderate stress, and scores 38–56 indicate severe stress). A Spanish validated version was used (26).

The PSQI was administered to assess the quality of sleep. It is a self-reported questionnaire that assesses sleep quality over an interval of 1 month. The overall score ranges between 0 and 21 and is the sum of seven components (sleep latency, subjective sleep quality, duration of sleep and sleep disturbances, habitual sleep efficiency, need of medication to sleep, and daytime dysfunction), with higher scores indicating poorer sleep quality, with an overall score of more than 5 indicating a “poor” sleeper. A Spanish validated version of the PSQI was used (27).

Sleep quality was also evaluated by actigraphy (ActiGraph wGT3X-BT accelerometer, ActiGraph, Pensacola, FL, United States) and the following variables were recorded: sleep latency, sleep efficiency, total time in bed, total sleep time, wakefulness after sleep onset, number of awakenings, and average number in minutes of awakenings.

Variables related to bias control were considered during the study, including body composition and physical activity. Body composition was analyzed by bioelectrical impedance analysis (BIA) on a whole-body BIA analyzer Tanita BC-420MA (Tanita Corporation, Tokyo, Japan). Variables analyzed included weight (kg), BMI (kg/m2), fat mass (kg), percentage of fat mass, and muscle mass (kg). The physical activity level was measured with the same accelerometer used to assess sleep quality. Assessments were conducted both at the study’s outset and after 12 weeks of product consumption. The evaluation period for the accelerometer was 3 weekdays and 1 weekend day, aiming to provide a weekly average. METs (metabolic equivalents) were used as the variable to determine physical activity levels, with 1 MET corresponding to the minimum oxygen consumption required to maintain vital functions, serving as a unit to compare the energy cost of daily activities.

### 2.6 Safety

The occurrence of adverse events (AEs) was monitored throughout the study by the investigators and based on subjects’ diary entries. Investigators rated the reported AEs as being either severe or non-severe based on their potential relationship to study treatment.

A blood analysis was conducted to determine the values of enzymes such as GOT (Glutamic Oxaloacetic Transaminase), GPT (Glutamic Pyruvic Transaminase GGT) (Gamma-Glutamyl Transferase), LDH (Lactate Dehydrogenase), and bilirubin for the assessment of liver function, as well as biomolecules like urea and creatinine to evalu-ate renal function. Additionally, a complete blood count (hemogram) was performed to assess red and white blood cell series, as well as platelets. Blood samples were obtained under fasting conditions at baseline and after 12 weeks of product consumption.

### 2.7 Statistical analysis

Frequencies and percentages were used for the expression of categorical variables, and mean ± standard deviation (SD) for continuous variables. The chi-square test or the Fisher’s exact test was used for the comparison of categorical variables between the study groups, and the Student’s t test for the comparison of quantitative variables. The analysis of variance (ANOVA) for repeated measures was used to assess the change of variables corresponding to each group throughout the study period. Within subject factor included data at baseline and at 8 weeks, and between-subject factor for paired data included the product administered that is, medicinal plant extract high dose, medicinal plant extract low dose, and placebo. The Turkey’s or Bonferroni’s correction was applied for post-hoc analyses. In the evaluation of changes in the Cornell Musculoskeletal Discomfort Questionnaire, patients who determined that they had no pain during the entire study in the indicated area of the body (score 0) were not included in the analysis. In order to assess the effect of the study product in subjects with minimal depression, anxiety, and perceived stress symptoms at the beginning of the study, secondary analyses were also performed using cut-points of ≥4 for BDI-II, ≥ 14 for STAI-state, and ≥ 16 for PSS. A *p* < 0.05 was considered statistically significant. Data analyses were performed with the SPSS version 25.0 (IBM Corp., Armonk, NY, United States) software program.

## 3 Results

### 3.1 General characteristics of participants

**Figure 1:**
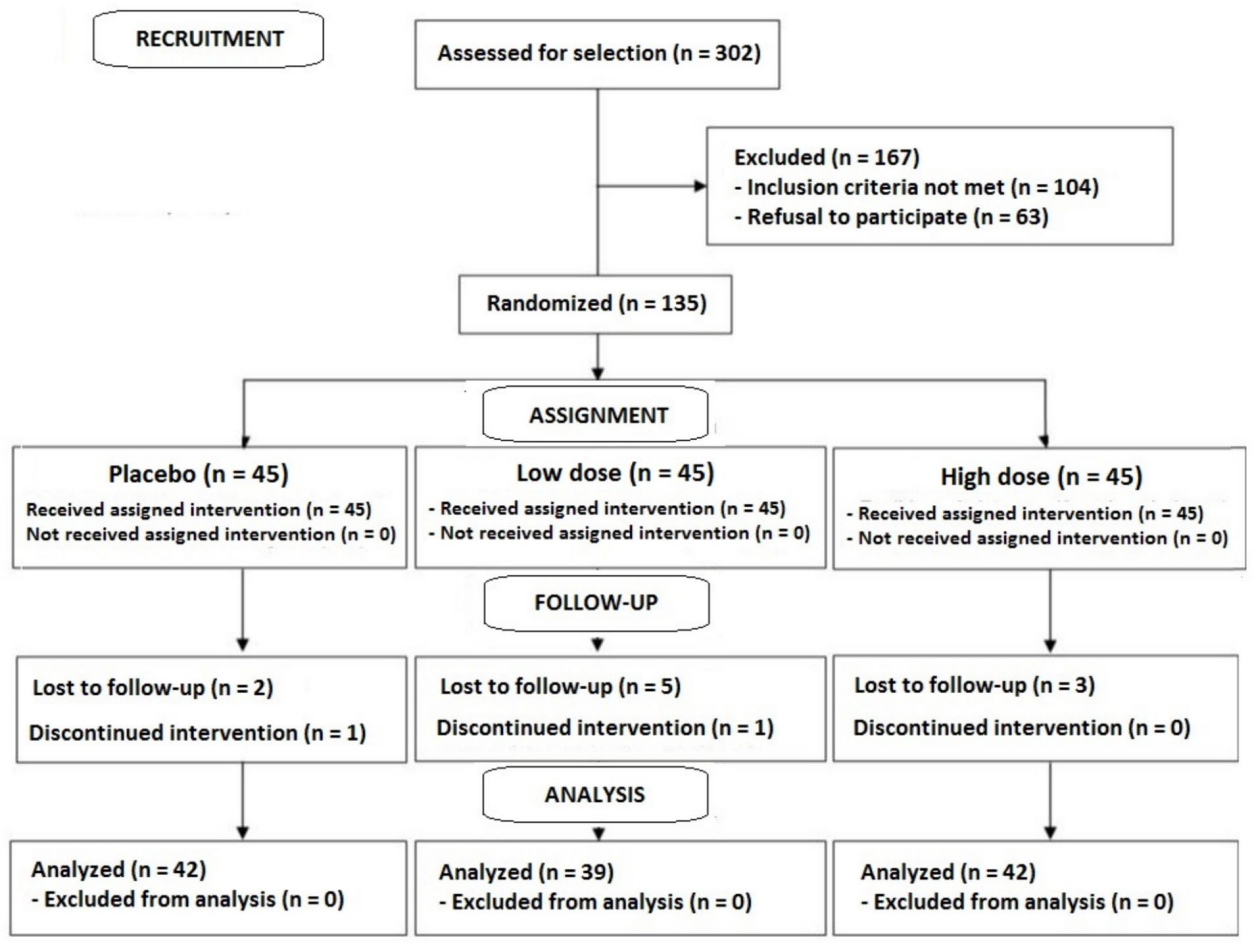
Flow chart of the study population.

A total of 302 subjects were initially selected, 135 of which were eligible, but 167 were excluded because the selection criteria were not met (*n* = 104) or refusal to participate (*n* = 63). The 135 eligible subjects were randomized 45 in each of the three study groups. However, 12 subjects were lost to follow-up (3 in the placebo group, 6 in the low dose group, and 3 in the high dose group). The analysis was finally carried out in 123 subjects (42 in the placebo group, 39 in the low dose group, and 42 in the high dose group). The flow chart of the study population is shown in Figure 1.

The study population included 43 men and 80 women, with a mean age of 30.9 ± 12.0 years. The mean VAS score of pain was 5.7 ± 1.4. Differences in demographic and clinical data at baseline between the study groups were not found (Table 1).

**Table 1:** Demographic and clinical data at baseline.

Variables	Placebo (*n* = 42)	Investigational product		Total (*n* = 123)
		Low dose (*n* = 39)	High dose (*n* = 42)	
Age, years	33.1 ± 12.2	31.4 ± 13.4	28.2 ± 10.0	30.9 ± 12.0
Weight, kg	70.5 ± 14.7	68.5 ± 13.2	67.6 ± 12.1	68.9 ± 13.3
BMI, kg/m2	24.1 ± 3.6	23.5 ± 3.4	23.6 ± 3.2	23.7 ± 3.4
Systolic BP, mmHg	115.2 ± 14.6	115.0 ± 13.3	113.5 ± 13.5	114.6 ± 13.8
Diastolic BP, mmHg	73.7 ± 9.0	74.0 ± 8.1	74.9 ± 9.3	74.2 ± 8.8
VAS score	5.7 ± 1.4	5.7 ± 1.4	5.9 ± 1.4	5.8 ± 1.4

Data as mean ± standard deviation; BMI, body mass index; BP, blood pressure; VAS, visual analogue scale.

### 3.2 Pain intensity

#### 3.2.1 Monthly VAS scores

In all study groups, VAS scores of pain intensity showed a statistically significant decrease at the end of the study as compared to baseline, but within-group differences were of greater magnitude in subjects assigned to the investigational product groups (Table 2). Also, between-group analyses showed statistically significant differences of VAS scores of the two groups of the investigational product as compared to placebo. The differences were noticeable from the second visit, i.e., from 28 days of product consumption (*p* < 0.006 dose-low; *p* < 0.001 dose-high). At the 56-day visit, the decrease was not as pronounced as in the early days of consumption, but significant differences persisted between the experimental products and the placebo (*p* < 0.050 dose-low; *p* < 0.017 dose-high). These significant differences remained at the end of the study (*p* < 0.044 dose-low; *p* < 0.005 dose-high).

**Table 2:** VAS scores of pain intensity at baseline and throughout the study period.

Study groups	VAS score, mean ± SD				Within- group *p* value	Between- group *p* value
	Visit 1 baseline	Visit 2 28 days	Visit 3 56 days	Visit 4 (final) 84 days		
Placebo (*n* = 42)	5.7 ± 1.4	4.7 ± 1.7	4.1 ± 2.1	3.7 ± 1.9	0.001	< 0.001
Investigational product						
Low dose (*n* = 39)	5.6 ± 1.4	3.4 ± 2.2	3.0 ± 2.5	2.5 ± 2.1	0.001	
High dose (*n* = 42)	5.9 ± 1.5	3.4 ± 2.0	3.0 ± 2.3	2.4 ± 2.4	0.001	

VAS, visual analogue scale; SD, standard deviation.

**Figure 2:**
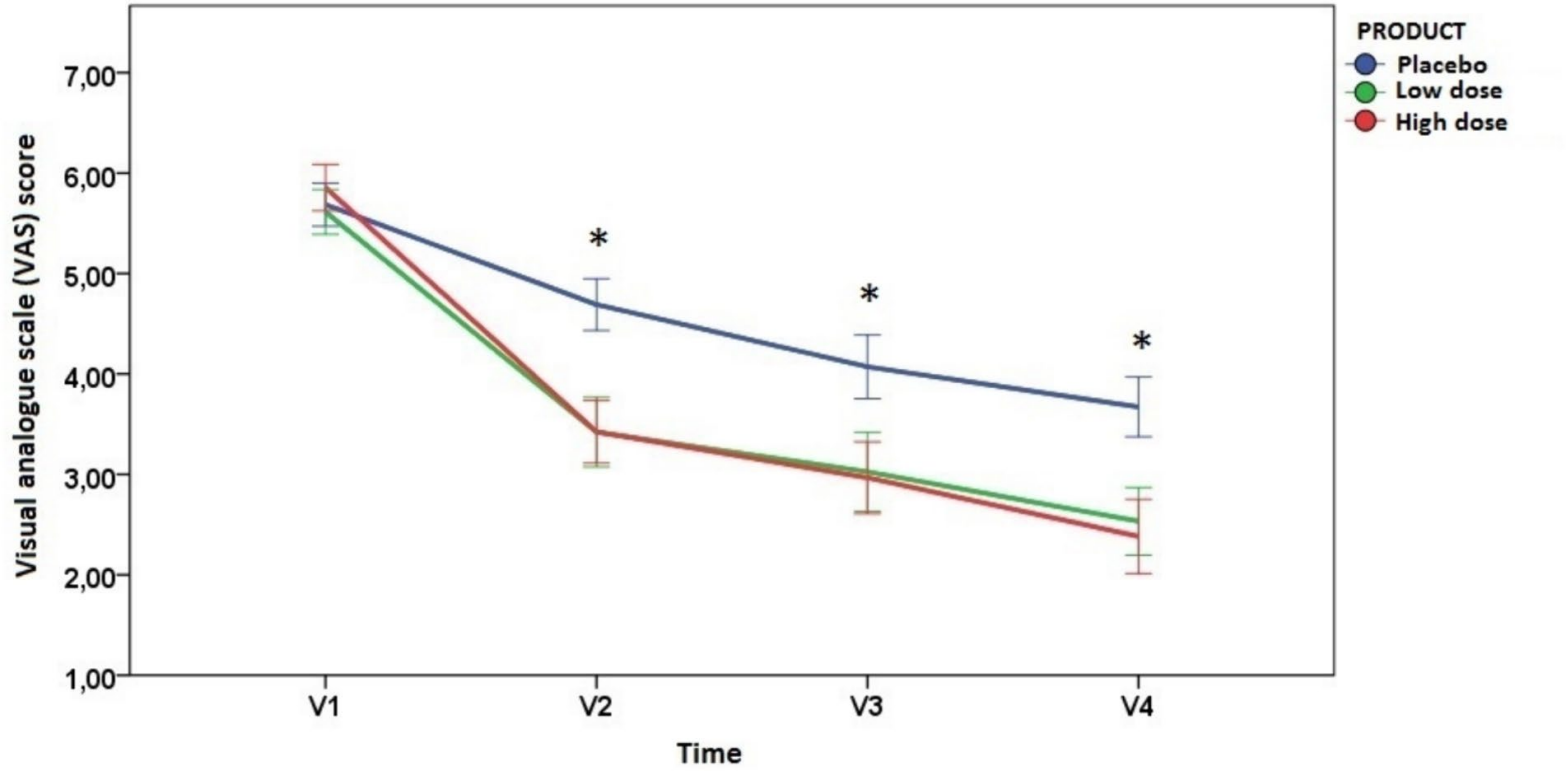
Changes of VAS scores during the study period (error bars ±1 standard deviation) (^*^indicates statistically significant differences between the placebo group and the investigational product groups; see text for *p* values at each time points).

At the end of the study (day 84) both experimental product doses led to a more significant and sustained reduction in pain compared to the placebo, with the low-dose product showing a reduction of approximately 56%, and the high-dose product demonstrating a reduction of about 59% as compared to their corresponding baseline values. The evolution of VAS scores over the study period is shown in Figure 2.

#### 3.2.2 Weekly VAS scores

**Figure 3:**
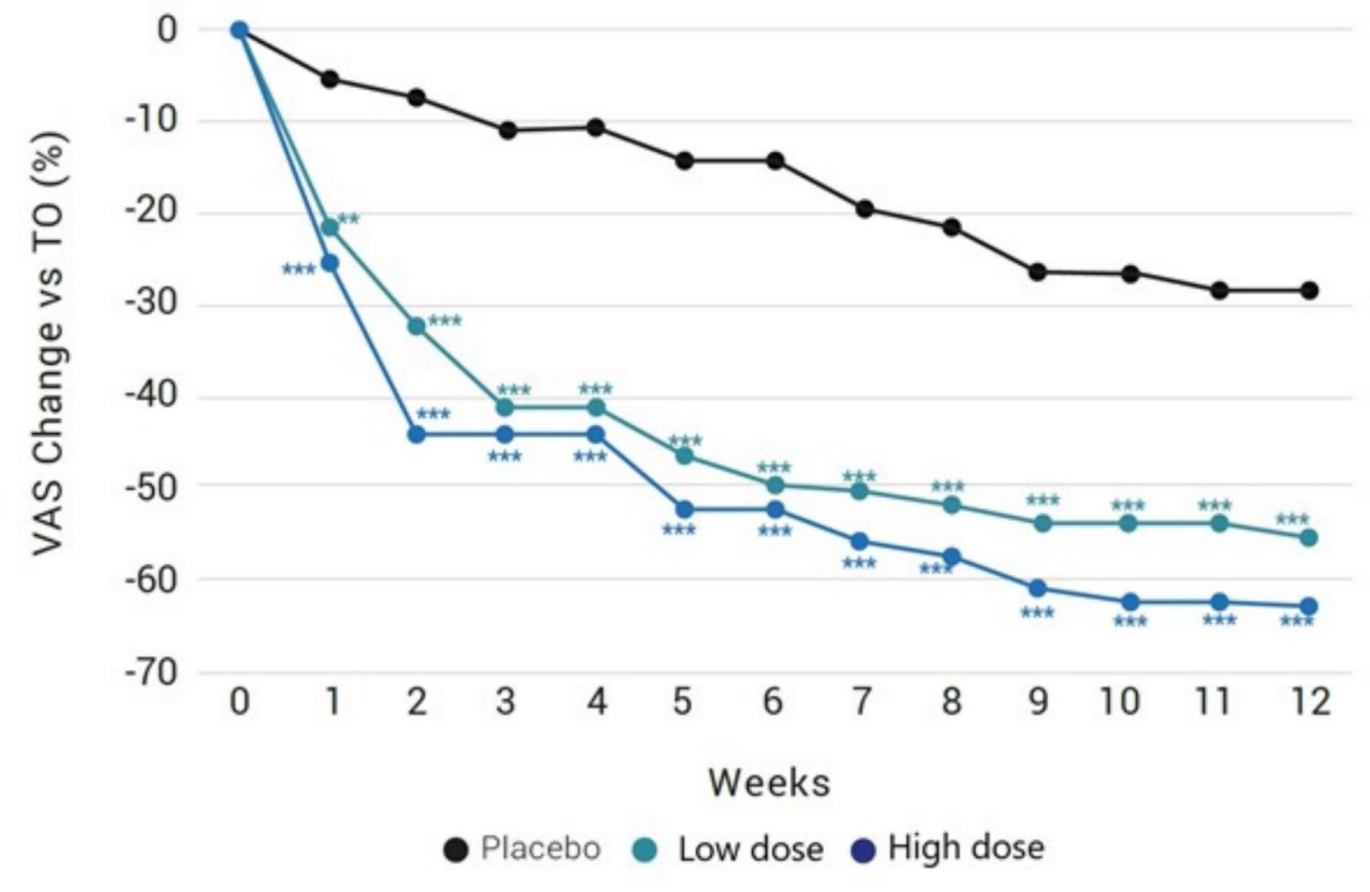
Weekly percentage changes of VAS scores in the study groups. ^*^Significant differences in comparison with placebo.

Results obtained in weekly VAS scores of pain intensity were similar than changes observed at the monthly study visits (Figure 3 and Table 3). In the placebo group, there were statistically significant within-group differences from the seventh week until the end of the study (28% VAS reduction), while in the two groups of the investigational product, statistically significant within-group differences were already observed from the first week reaching a 55.3 and 62.7% reduction for the low and high dose, respectively, from baseline by the end of the 12-week period.

**Table 3:** Weekly changes of VAS scores of pain intensity in the three study groups.

Time point	Study groups, VAS score, mean ± SD		
	Placebo (*n* = 42)	Investigational product	
		Low dose (*n* = 39)	High dose (*n* = 42)
Baseline	5.7 ± 1.4	5.6 ± 1.4	5.9 ± 1.5
Week 1	5.4 ± 1.6	4.4 ± 1.8	4.4 ± 1.6
Week 2	5.3 ± 1.6	3.8 ± 1.8	3.3 ± 1.6
Week 3	5.3 ± 1.7	3.3 ± 1.8	3.3 ± 1.6
Week 4	5.1 ± 1.7	3.3 ± 2.0	3.4 ± 1.7
Week 5	4.9 ± 1.8	3.0 ± 2.1	2.8 ± 2.0
Week 6	4.9 ± 1.8	2.8 ± 2.0	2.8 ± 1.9
Week 7	4.6 ± 1.8	2.8 ± 2.1	2.6 ± 1.9
Week 8	4.5 ± 1.8	2.7 ± 2.1	2.5 ± 1.8
Week 9	4.2 ± 1.9	2.6 ± 2.1	2.3 ± 2.0
Week 10	4.2 ± 1.8	2.6 ± 2.0	2.2 ± 2.0
Week 11	4.1 ± 1.8	2.6 ± 2.1	2.2 ± 2.0
Week 12	4.1 ± 1.8	2.5 ± 2.0	2.2 ± 2.2
Within-group *p* value	0.015 (from week 7)	< 0.001 (from week 1)	< 0.001 (from week 1)
Between-group *p* value	< 0.001		

SD, standard deviation.

On the other hand, there were between-group statistically significant differences of VAS scores of the two groups of the investigational product as compared to placebo from the first week of treatment until the end. Differences between the low and high dose groups of the investigational product were not found (*p* = 0.726 at the end of the study).

### 3.3 Analgesic medication

At the beginning of the study, 70 (56.9%) participants [placebo group 22 (31.4%), experimental group-low dose 23 (32.9%), experimental group-high dose 25 (35.7%)] used some analgesic medication for the relief of back pain. Medications initially consumed were ibuprofen (24.4%), paracetamol (17%), dexketoprofen trometamol (12.2%), metamizole magnesium (4.9%), naproxen (3.3%), and diclofenac (1.6%). Local heat pads were used by 12.2% of subjects. Throughout the study, the modification of these treatments was monitored, revealing a decrease in consumption across all study groups after 28 days (Table 4). Apart from identifying notable variations in treatment reduction during each study visit, both experimental groups exhibit considerable statistical significance when compared to placebo group (*p* < 0.001) from the first visit. Regarding the low dose and high dose groups, only significant differences were detected only in the first visit (28 days *p* < 0.043; 56 days *p* = 0.077; 84 days *p* = 0.330). At the end of the study, most of the subject in the experimental groups reduced their medication/heat treatment (87% in the low dose and 96% in the high dose group) (Table 4).

**Table 4:** Percentage of subjects who decreased medication/heat consumption compared to the baseline state for each of the study groups.

Study groups	Medication/heat (% decrease)			Within- group *p* value	Between- group *p* value
	Visit 2 28 days	Visit 3 56 days	Visit 4 (final) 84 days		
Placebo (*n* = 42)	4.5%	13.6%	27.3%	0.001	< 0.001
Investigational product					
Low dose (*n* = 39)	47.8%	78.3%	87%	0.001	
High dose (*n* = 42)	76%	96%	96%	0.001	

### 3.4 Cornell musculoskeletal discomfort questionnaire

Results obtained of the Cornell questionnaire in the different body areas are shown in Table 5. The two doses of the investigational product were more effective than placebo in the relief of the level of discomfort in all five body areas, especially in the neck and back pain, which were the variables used for the inclusion of the subjects. However, despite the clear trend of improvement with both doses of the product, statistically significant differences compared to the start of the study were observed in all areas for the high dose and only in the neck and back pain for the low dose. The reason for this could be attributed to the fact that there were very few participants with shoulder pain. Additionally, subjects taking the low dose started with lower pain values, particularly in subjects with left shoulder pain. Between-group significant differences were found in the analysis of neck and upper back areas, with a lower level of discomfort in the investigational product groups as compared to placebo.

**Table 5:** Changes in the Cornell musculoskeletal discomfort questionnaire in the three study groups.

Body areas and study groups	Mean ± SD scores				Within- group *p* value	Between- group *p* value
	Visit 1 baseline	Visit 2 28 days	Visit 3 56 days	Visit 4 (final) 84 days		
Neck						
Placebo (*n* = 34)	16.7 ± 25.0	11.0 ± 11.5	10.9 ± 17.7	10.1 ± 11.2	0.688	0.028
Low dose (*n* = 34)	23.4 ± 24.2	11.5 ± 14.1^*^	10.1 ± 13.6^*^	7.9 ± 12.3^*^	0.002	
High dose (*n* = 34)	29.0 ± 26.7	14.0 ± 19.5^*^	10.9 ± 18.8^*^	5.0 ± 15.7^*^	0.001	
Right shoulder						
Placebo (*n* = 17)	16.2 ± 21.9	10.6 ± 10.9	8.8 ± 15.8	9.7 ± 10.7	1.000	0.968
Low dose (*n* = 17)	11.2 ± 23.1	5.4 ± 9.9	5.1 ± 10.0	2.4 ± 4.7	0.396	
High dose (*n* = 22)	13.2 ± 15.8	6.7 ± 12.2	5.4 ± 9.2	1.6 ± 3.5^*^	0.040	
Left shoulder						
Placebo (*n* = 17)	10.1 ± 21.4	5.7 ± 7.4	4.8 ± 14.7	3.7 ± 7.0	1.000	0.316
Low dose (*n* = 14)	4.7 ± 7.0	4.1 ± 5.8	2.1 ± 3.9	3.0 ± 6.2	1.000	
High dose (*n* = 21)	16.6 ± 20.8	12.3 ± 20.3	9.0 ± 19.6	2.0 ± 3.8^*^	0.006	
Upper back						
Placebo (*n* = 33)	15.6 ± 19.2	13.0 ± 22.1	7.7 ± 12.3	8.1 ± 11.9	0.227	0.011
Low dose (*n* = 31)	15.7 ± 14.9	8.4 ± 10.4	6.3 ± 16.6	3.3 ± 6.3^*^	0.007	
High dose (*n* = 33)	27.3 ± 26.4	10.0 ± 14.3^*^	7.5 ± 9.5^*^	3.9 ± 9.1^*^	0.001	
Lower back						
Placebo (*n* = 34)	12.1 ± 16.7	10.8 ± 20.6	9.7 ± 18.5	11.0 ± 22.6	1.000	0.201
Low dose (*n* = 33)	17.4 ± 20.6	12.6 ± 16.6	10.3 ± 16.6	7.1 ± 10.4^*^	0.049	
High dose (*n* = 33)	16.3 ± 20.3	10.9 ± 11.5	5.2 ± 5.8^*^	4.0 ± 7.2^*^	0.018	

SD, standard deviation. ^*^Statistically significant compared to the baseline. Patients with no pain at baseline were excluded from the analysis.

### 3.5 Pain-related disability

Baseline scores of the RMD questionnaire were not homogeneous among the three study groups, with higher scores in the high dose group (4.9 ± 2.8) compared to the low dose (3.9 ± 2.6) (*p* = 0.197) and the placebo group (3.2 ± 2.1) (*p* < 0.007). Scores of the RMD questionnaire decreased significantly in all groups throughout the study, as well as significant within-group differences, with greater improvements in the investigational product groups (Table 6). However, clinically relevant changes (≥ 2 points reduction) were only found in the groups treated with the supplement ingredient (in the low dose −2.3 points and high dose −2.9 points).

**Table 6:** Changes in the Roland-Morris Disability (RMD) questionnaire in the three study groups.

Study groups	RMD points, mean ± SD				Within- group *p* value	Between- group *p* value
	Visit 1 baseline	Visit 2 28 days	Visit 3 56 days	Visit 4 (final) 84 days		
Placebo (*n* = 42)	3.2 ± 2.1	2.4 ± 1.9^*^	2.3 ± 2.2^*^	2.0 ± 2.6^*^	0.009	0.003
Investigational product						
Low dose (*n* = 39)	3.9 ± 2.6	2.7 ± 2.0^*^	2.3 ± 1.8^*^	1.6 ± 1.9^*^	0.001	
High dose (*n* = 42)	4.9 ± 2.8	3.5 ± 2.5^*^	2.6 ± 2.4^*^	2.0 ± 2.5^*^	0.001	

SD, standard deviation. ^*^Statistically significant compared to baseline.

### 3.6 Quality of life

#### 3.6.1 Quality of life assessed with the SF-36 questionnaire

As shown in Table 7, statistically significant differences favoring the investigational product in both low and high dose doses compared to placebo were found in the domains of bodily pain and the health transition question, which coincidently were the domains with the lowest starting average scores. In the domains of physical functioning, physical role limitations, and bodily pain, within-group values were statistically significant in all three study groups, whereas significant improvements in energy/vitality and health transition question were found in both investigational product groups. Additionally, significant increases in emotional role and social functioning were observed specifically in the high-dose group.

**Table 7:** Changes in quality of life in the three study groups according to the SF-36.

SF-36 domains and study groups	Mean ± SD scores				Within- group *p* value	Between- group *p* value
	Visit 1 baseline	Visit 2 28 days	Visit 3 56 days	Visit 4 (final) 84 days		
Physical functioning						
Placebo (*n* = 42)	84.8 ± 13.3	88.3 ± 13.0	89.3 ± 12.1^*^	90.2 ± 9.4^*^	0.006	0.814
Low dose (*n* = 39)	86.4 ± 11.0	89.1 ± 9.7	92.4 ± 7.4^*^	93.3 ± 6.2^*^	0.001	
High dose (*n* = 42)	83.2 ± 16.1	85.0 ± 20.4	88.6 ± 14.5^*^	90.4 ± 14.0^*^	0.001	
Physical role limitations						
Placebo (*n* = 42)	73.2 ± 18.7	81.9 ± 15.9^*^	81.0 ± 18.4^*^	82.4 ± 16.1^*^	0.006	0.503
Low dose (*n* = 39)	70.4 ± 18.1	80.0 ± 19.0^*^	82.4 ± 18.0^*^	85.1 ± 14.9^*^	0.001	
High dose (*n* = 42)	68.7 ± 21.7	76.0 ± 21.4^*^	77.5 ± 19.4^*^	82.7 ± 18.9^*^	0.001	
Bodily pain						
Placebo (*n* = 42)	47.5 ± 16.1	55.1 ± 16.6^*^	54.7 ± 18.2^*^	56.4 ± 17.2^*^	0.018	0.025
Low dose (*n* = 39)	50.0 ± 15.0	56.3 ± 15.6	60.2 ± 18.6^*^	65.6 ± 20.1^*^	0.001	
High dose (*n* = 42)	46.5 ± 16.4	58.4 ± 16.0^*^	61.1 ± 18.1^*^	67.8 ± 21.7^*^	0.001	
General health perception						
Placebo (*n* = 42)	68.3 ± 15.4	69.8 ± 14.2	71.2 ± 14.3	70.6 ± 14.2	1.000	0.998
Low dose (*n* = 39)	66.1 ± 21.1	68.8 ± 19.8	69.4 ± 20.4	70.0 ± 22.1	0.642	
High dose (*n* = 42)	68.0 ± 19.3	70.7 ± 16.1	71.2 ± 16.6	71.7 ± 16.7	0.642	
Energy/vitality						
Placebo (*n* = 42)	55.8 ± 15.8	61.3 ± 17.2	61.8 ± 16.8	62.1 ± 14.4	0.112	0.823
Low dose (*n* = 39)	55.0 ± 16.9	59.8 ± 15.9	63.3 ± 17.0^*^	64.6 ± 19.7^*^	0.003	
High dose (*n* = 42)	55.5 ± 17.4	62.4 ± 16.5^*^	63.8 ± 14.8^*^	65.6 ± 14.8^*^	0.001	
Social functioning						
Placebo (*n* = 42)	76.0 ± 21.8	77.7 ± 19.8	79.5 ± 21.5	80.1 ± 21.4	1.000	0.888
Low dose (*n* = 39)	79.2 ± 19.7	84.0 ± 15.7	84.3 ± 20.6	86.5 ± 17.5	0.107	
High dose (*n* = 42)	74.2 ± 21.2	81.3 ± 19.2	80.7 ± 20.7	82.4 ± 19.1^*^	0.037	
Emotional role						
Placebo (*n* = 42)	80.6 ± 18.4	85.1 ± 18.4	84.5 ± 18.3	84.5 ± 20.0	1.000	0.216
Low dose (*n* = 39)	80.3 ± 20.7	82.3 ± 19.6	85.5 ± 17.2	84.0 ± 23.2	0.350	
High dose (*n* = 42)	73.8 ± 22.9	82.2 ± 22.2^*^	85.1 ± 19.0^*^	85.7 ± 19.2^*^	0.002	
Mental health						
Placebo (*n* = 42)	70.7 ± 15.2	74.5 ± 15.4	73.0 ± 18.5	74.5 ± 18.0	0.800	0.866
Low dose (*n* = 39)	71.7 ± 14.8	73.6 ± 16.7	75.3 ± 15.9	75.5 ± 17.7	0.846	
High dose (*n* = 42)	71.7 ± 20.0	73.6 ± 18.2	73.0 ± 16.3	76.8 ± 17.7	0.257	
Health transition question						
Placebo (*n* = 42)	51.8 ± 20.2	49.4 ± 16.1	52.4 ± 14.4	50.0 ± 12.3	1.000	0.023
Low dose (*n* = 39)	46.2 ± 15.7	54.5 ± 21.4^*^	53.2 ± 21.6^*^	56.4 ± 24.1^*^	0.033	
High dose (*n* = 42)	49.4 ± 21.7	58.7 ± 22.9^*^	59.5 ± 23.4^*^	59.5 ± 24.7^*^	0.027	

SD, standard deviation. ^*^Statistically significant compared to baseline.

#### 3.6.2 PROMIS-29 questionnaire

As shown in Table 8, there was a statistically significant general improvement in all domains compared to baseline in the groups taking the investigational product. In the placebo group, improvements over time were only observed in the pain interference, pain intensity, and anxiety domains, although the magnitude of improvements was higher in the two doses of the investigational product. Between group statistically significant differences were only found for the item of pain intensity from the second visit, with higher decreases in the investigational product groups. No significant differences were found among the two dosages studied in the pain intensity item.

**Table 8:** Changes in the PROMIS-29 questionnaire in the three study groups.

Domains and study groups	Mean ± SD scores				Within- group *p* value	Between- group *p* value
	Visit 1 baseline	Visit 2 28 days	Visit 3 56 days	Visit 4 (final) 84 days		
Pain interference						
Placebo (*n* = 42)	15.0 ± 3.0	16.5 ± 2.9^*^	16.8 ± 3.0^*^	17.2 ± 3.0^*^	0.001	0.296
Low dose (*n* = 39)	14.6 ± 3.4	16.4 ± 2.9^*^	17.3 ± 3.0^*^	18.0 ± 2.4^*^	0.001	
High dose (*n* = 42)	13.7 ± 3.5	15.6 ± 3.8^*^	16.6 ± 3.2^*^	17.5 ± 3.4^*^	0.001	
Physical function						
Placebo (*n* = 42)	18.4 ± 1.7	18.9 ± 1.9^*^	19.0 ± 1.7	18.6 ± 2.8	1.000	0.231
Low dose (*n* = 39)	18.7 ± 1.9	19.2 ± 1.1^*^	19.4 ± 1.3^*^	19.4 ± 1.0^*^	0.049	
High dose (*n* = 42)	18.3 ± 2.4	18.9 ± 1.9^*^	19.0 ± 1.7^*^	19.4 ± 1.6^*^	0.004	
Pain intensity						
Placebo (*n* = 42)	5.8 ± 1.4	5.0 ± 1.4^*^	4.6 ± 1.9^*^	4.2 ± 1.8^*^	0.001	0.002
Low dose (*n* = 39)	5.5 ± 1.5	4.3 ± 1.8^*^	3.6 ± 2.1^*^	3.2 ± 2.2^*^	0.001	
High dose (*n* = 42)	5.9 ± 1.5	4.1 ± 1.7^*^	3.5 ± 2.0^*^	2.9 ± 2.0^*^	0.001	
Anxiety						
Placebo (*n* = 42)	15.6 ± 3.4	16.3 ± 2.7	16.6 ± 3.3	17.0 ± 3.1^*^	0.013	0.402
Low dose (*n* = 39)	15.1 ± 3.3	16.2 ± 3.1^*^	16.7 ± 3.3^*^	17.8 ± 2.5^*^	0.001	
High dose (*n* = 42)	15.0 ± 3.7	16.0 ± 3.4^*^	16.4 ± 2.9^*^	17.3 ± 2.8^*^	0.001	
Depression						
Placebo (*n* = 42)	18.0 ± 2.8	18.4 ± 2.5	18.6 ± 2.4	18.8 ± 2.1	0.507	0.798
Low dose (*n* = 39)	17.7 ± 3.2	17.9 ± 3.3	18.6 ± 2.0^*^	19.0 ± 1.4^*^	0.002	
High dose (*n* = 42)	17.8 ± 2.6	18.3 ± 2.6	18.7 ± 1.9	19.1 ± 1.5^*^	0.003	
Fatigue						
Placebo (*n* = 42)	15.1 ± 2.6	16.2 ± 2.7	16.0 ± 2.8	16.1 ± 3.1	0.118	0.534
Low dose (*n* = 39)	14.5 ± 3.6	15.5 ± 2.8	15.9 ± 3.0^*^	16.6 ± 3.4^*^	0.001	
High dose (*n* = 42)	14.2 ± 4.0	14.9 ± 4.2	15.4 ± 3.2^*^	15.7 ± 3.7^*^	0.012	
Sleep disturbance						
Placebo (*n* = 42)	13.5 ± 2.8	14.1 ± 2.7	14.0 ± 3.1	14.2 ± 2.9	1.000	0.797
Low dose (*n* = 39)	13.3 ± 3.8	14.3 ± 3.4	14.7 ± 2.9^*^	14.6 ± 2.9^*^	0.031	
High dose (*n* = 42)	13.4 ± 3.2	14.3 ± 2.3	14.7 ± 2.5^*^	15.0 ± 2.8^*^	0.002	
Satisfaction with social roles						
Placebo (*n* = 42)	15.0 ± 3.7	15.5 ± 3.2	15.4 ± 3.0	15.6 ± 2.8	1.000	0.812
Low dose (*n* = 39)	14.9 ± 3.9	15.5 ± 3.6	16.0 ± 2.9	16.2 ± 3.0^*^	0.049	
High dose (*n* = 42)	14.5 ± 4.3	14.9 ± 3.7	15.1 ± 3.8	15.9 ± 3.3^*^	0.035	

SD, standard deviation. ^*^Statistically significant compared to baseline.

The high dose group also showed greater increases in physical function at the end of the study compared to placebo (*p* < 0.05).

#### 3.6.3 Emotional wellbeing

Emotional wellbeing was evaluated through different validated questionaries: Beck Depression Inventory (BDI), State–Trait Anxiety Inventory (STAI), and Perceived Stress Scale (PSS). These renowned questionnaires collectively provide valuable in-sights into various dimensions of mental health, offering a nuanced understanding of an individual’s emotional state. The results of these questionaries are included in Tables 9, 10.

**Table 9:** Changes of emotional well-being dimensions in the three study groups.

Questionnaires and study groups	Mean ± SD scores				Within- group *p* value	Between- group *p* value
	Visit 1 baseline	Visit 2 28 days	Visit 3 56 days	Visit 4 (final) 84 days		
BDI-II score						
Placebo (*n* = 42)	7.4 ± 4.8	6.0 ± 4.5	6.3 ± 5.0	6.1 ± 5.1	0.281	0.149
Low dose (*n* = 39)	7.3 ± 5.1	5.9 ± 4.7	5.4 ± 5.0^*^	4.8 ± 5.1^*^	0.002	
High dose (*n* = 42)	8.8 ± 6.4	7.5 ± 7.2	6.0 ± 6.6^*^	5.8 ± 5.9^*^	0.001	
STAI-state, score						
Placebo (*n* = 42)	17.3 ± 9.4	17.6 ± 8.3	17.0 ± 10.2	16.4 ± 10.3	1.000	0.232
Low dose (*n* = 39)	17.2 ± 9.9	16.8 ± 10.9	15.8 ± 10.5	14.6 ± 10.6	0.349	
High dose (*n* = 42)	17.7 ± 10.9	16.3 ± 11.0	15.1 ± 10.7	12.4 ± 9.2^*^	0.001	
STAI-trait, score						
Placebo (*n* = 42)	19.1 ± 10.0	17.0 ± 8.9	16.2 ± 10.9^*^	16.2 ± 10.4^*^	0.038	0.480
Low dose (*n* = 39)	17.4 ± 9.1	16.9 ± 9.2	15.0 ± 9.4	15.0 ± 8.9^*^	0.049	
High dose (*n* = 42)	19.3 ± 10.7	16.4 ± 8.9^*^	15.6 ± 10.0^*^	14.6 ± 10.2^*^	0.001	
PSS total score						
Placebo (*n* = 42)	20.2 ± 7.7	20.1 ± 7.8	19.5 ± 8.6	18.7 ± 7.8	1.000	0.328
Low dose (*n* = 39)	19.8 ± 8.8	18.6 ± 8.6	17.9 ± 9.0	17.1 ± 9.5^*^	0.049	
High dose (*n* = 42)	20.9 ± 8.5	18.5 ± 9.3^*^	18.2 ± 9.3^*^	16.2 ± 8.9^*^	0.001	

SD, standard deviation; BDI, beck depression inventory; STAI, state–trait anxiety inventory; PSS, perceived stress scale. ^*^Statistically significant compared to baseline.

**Table 10:** Changes of depression, anxiety, and perceived stress levels in the three study groups among subjects with minimal symptoms at the beginning of the study.

Questionnaires and study groups	Mean ± SD scores				Within- group *p* value	Between- group *p* value
	Visit 1 baseline	Visit 2 28 days	Visit 3 56 days	Visit 4 (final) 84 days		
BDI-II score ≥ 4						
Placebo (*n* = 35)	8.5 ± 4.4	6.9 ± 4.5	7.1 ± 5.1	6.8 ± 5.3	0.095	0.016
Low dose (*n* = 31)	8.9 ± 4.4	7.1 ± 4.6	6.5 ± 4.9^*^	5.8 ± 5.2^*^	0.001	
High dose (*n* = 32)	10.9 ± 6.1	8.9 ± 7.6^*^	6.7 ± 7.1^*^	6.4 ± 6.5^*^	0.001	
STAI-state, score ≥ 14						
Placebo (*n* = 25)	23.5 ± 6.6	21.8 ± 6.8	22.0 ± 9.7	21.9 ± 9.4	1.000	0.038
Low dose (*n* = 21)	24.6 ± 7.1	23.2 ± 9.7	20.2 ± 8.7	18.8 ± 8.1^*^	0.009	
High dose (*n* = 25)	24.3 ± 9.0	20.6 ± 11.0	19.0 ± 10.9^*^	15.6 ± 10.0^*^	0.001	
PSS score ≥ 16						
Placebo (*n* = 31)	23.6 ± 5.7	23.3 ± 6.3	22.8 ± 7.3	21.8 ± 6.2	1.000	0.018
Low dose (*n* = 24)	25.2 ± 6.8	23.0 ± 7.3	22.0 ± 8.3	21.5 ± 8.1^*^	0.048	
High dose (*n* = 28)	25.5 ± 6.2	21.8 ± 8.7^*^	20.1 ± 9.1^*^	18.0 ± 8.9^*^	0.001	

SD, standard deviation; BDI, beck depression inventory; STAI, state–trait anxiety inventory; PSS, perceived stress scale. ^*^Statistically significant compared to baseline.

The Beck questionnaire related to the state of depression revealed a decrease in all groups for each of the measurements conducted in relation to the initial moment. However, only significant differences compared to the start of the study were observed in the two doses of the investigational product. When comparing with placebo, no statistically significant differences were apparent; however, a trend towards significance was observed (*p* = 0.149) (Table 9). When conducting a more in-depth analysis using cut-points of ≥4 for BDI-II to identify subjects with minimal symptoms, significant differences in BDI-II scores were observed in investigational product groups compared to placebo (*p* = 0.016) (Table 10). This difference was more pronounced in the high-dose group, where significant differences compared to placebo were observed starting from the second visit (*p* < 0.05).

Although there was a significant reduction in anxiety levels within the high-dose group when compared to the beginning of the study, the investigational products were not significantly superior to placebo in improving anxiety within the overall study population, encompassing both state and trait aspects (Table 9). However, when considering only the subjects who reported an anxiety level of 14 or higher, as assessed by the STAI questionnaire, significant differences in progress were observed between the groups (*p* = 0.038), with the high-dose group exhibiting a noteworthy improvement after 84 days of product consumption (*p* < 0.008) (Table 10).

Finally, in the entire population, the investigational products were not superior to placebo in reducing the perceived stress levels as measured by PSS score (*p* = 0.328). However, in the low and high dose groups of the experimental product, some improvement was seen as compared to the baseline at the end of the study in the low dose group, and from the second visit in the high dose group (Table 9). Furthermore, when analyzing the population with minimal stress (PSS ≥ 16), significant differences in progress were observed between the groups (*p* = 0.018). Specifically, the high-dose group exhibited a significant improvement from the 56th day of product consumption (*p* < 0.05) that was maintained until the end of the study (*p* < 0.009) (Table 10).

#### 3.6.4 Sleep quality: perceived sleep quality index (PSQI) and actigraphy

Sleep quality was assessed both at the perception level and using a tracking de-vice. As shown in Table 11, only the group consuming a high dose of the investigational product revealed a statistically significant improvement in their perceived sleep quality index (PSQI) throughout the study period, starting from the 28th day (*p* < 0.05) until the end of the study (*p* < 0.001). Also, significant differences were observed be-tween experimental and placebo groups (*p* = 0.036), whereas these differences were more noticeable in the higher dose.

**Table 11:** Changes in the Pittsburgh Sleep Quality Index (PSQI) scores in the three study groups.

PSQI score and study groups	Mean ± SD scores				Within- group *p* value	Between- group *p* value
	Visit 1 baseline	Visit 2 28 days	Visit 3 56 days	Visit 4 (final) 84 days		
Placebo (*n* = 42)	6.5 ± 2.5	6.1 ± 2.6	6.2 ± 2.9	6.0 ± 3.2	1.000	0.036
Low dose (*n* = 39)	6.9 ± 3.3	6.5 ± 2.9	6.2 ± 2.2	6.0 ± 2.9	0.230	
High dose (*n* = 42)	7.5 ± 3.8	6.4 ± 3.4^*^	5.8 ± 3.0^*^	5.3 ± 3.1^*^	0.001	

SD, standard deviation; PSQI, Pittsburgh Sleep Quality Index. ^*^Statistically significant compared baseline.

The assessment of sleep quality by actigraphy (Table 12) revealed that in general the population of the study had good sleep quality, with low sleep latency, high sleep efficiency (> 90%) with a total sleep time around 7 h, etc. Despite this, it was observed that the groups taking the experimental product significantly reduced latency compared to the study’s outset. When comparing the groups, significant differences were observed in relation to the placebo group in the high-dose group (*p* < 0.001) and a trend towards significance was observed between the control and the low-dose groups (*p* = 0.107).

**Table 12:** Results of sleep evaluation by actigraphy in the three study groups at the end of the study as compared with baseline.

Sleep parameters and study groups	Mean ± SD		Within- group *p* value	Between- group *p* value
	Visit 1 baseline	Visit 4 (final) 84 days		
Sleep latency, min				
Placebo (*n* = 42)	3.16 ± 1.02	3.36 ± 0.89	0.203	0.001
Low dose (*n* = 39)	3.37 ± 0.93	3.07 ± 0.82	0.049^*^	
High dose (*n* = 42)	3.53 ± 0.90	2.81 ± 0.84	0.001^*^	
Sleep efficiency, %				
Placebo (*n* = 42)	92.0 ± 3.4	91.2 ± 3.6	0.049	0.106
Low dose (*n* = 39)	91.8 ± 2.6	91.8 ± 3.1	0.996	
High dose (*n* = 42)	91.5 ± 3.3	92.0 ± 3.0	0.246	
Total time in bed, min				
Placebo (*n* = 42)	464.0 ± 53.9	461.1 ± 49.4	0.697	0.680
Low dose (*n* = 39)	446.1 ± 62.9	448.7 ± 58.7	0.736	
High dose (*n* = 42)	463.7 ± 61.1	469.9 ± 57.0	0.399	
Total sleep time, min				
Placebo (*n* = 42)	427,0 ± 51,2	420,9 ± 49,8	0.405	0.387
Low dose (*n* = 39)	409,4 ± 58,3	411,9 ± 56,2	0.735	
High dose (*n* = 42)	424,5 ± 60,1	432,5 ± 56,1	0.272	
Wakefulness after sleep onset, min				
Placebo (*n* = 42)	33.9 ± 16.3	36.9 ± 16.5	0.150	0.380
Low dose (*n* = 39)	33.4 ± 13.7	33.7 ± 14.0	0.875	
High dose (*n* = 42)	35.6 ± 14.8	34.6 ± 14.0	0.622	
Number of awakenings				
Placebo (*n* = 42)	14.5 ± 6.0	15.5 ± 6.8	0.190	0.204
Low dose (*n* = 39)	14.7 ± 5.5	14.8 ± 5.5	0.929	
High dose (*n* = 42)	14.4 ± 5.2	13.5 ± 6.0	0.224	
Awakenings, mean number of min				
Placebo (*n* = 42)	2.35 ± 0.78	2.57 ± 0.95	0.123	0.419
Low dose (*n* = 39)	2.36 ± 0.84	2.23 ± 0.69	0.808	
High dose (*n* = 42)	2.62 ± 1.19	2.79 ± 1.28	0.230	

SD, standard deviation; min, minutes. ^*^Statistically significant compared to baseline.

In the other actigraphy evaluated parameters, although a trend towards improvement was observed in sleep efficiency and number of awakenings in favor of the investigational product, it did not reach statistical significance.

### 3.7 Compliance and safety

The percentage of compliance ranged between 94 and 100% (some subjects re-turned 10 unconsumed capsules). Changes in the level of physical activity were not significant in any study group. At baseline and at the end of study, the mean values were 1.5 ± 0.19 and 1.54 ± 0.23 METs in the placebo group (*p* = 0.257), 1.54 ± 0.02 and 1.57 ± 0.24 METs in the low-dose group (*p* = 0.238), 1.50 ± 0.22 and 1.53 ± 0.24 METs in the high-dose group (*p* = 0.221). In addition, changes in BMI, percentage of fat mass, and SBP and DBP during the study period were not observed.

Regarding the safety, the results of physical examination were unrevealing and laboratory tests remained within the normal ranges in the three groups, with no significant changes in the blood count or on the liver or kidney function.

Adverse events of mild to moderate intensity or unrelated to the study product were recorded. Adverse events of mild to moderate intensity included stomach discomfort (placebo 7.1% of patients, low dose 15.4%, high dose 16.7%) and constipation (placebo 2.4% of patients, low dose 4.8%, high dose 4.5%), but none of these symptoms was considered a risk factor for discontinuation of dietary supplementation.

## 4 Discussion

In this study, the effect of a dietary supplement ingredient comprised of rosemary leaf, ashwagandha root, and sesame seed, administered in low and high doses of 400 and 800 mg/day, was assessed on a population of individuals with persistent myofascial back pain for 12 weeks. For that purpose, a randomized, double-blind placebo controlled clinical trial was conducted. The results of the study showed that the botanical extract blend was effective in reducing the intensity of back myofascial pain, to a significantly greater extent compared to placebo. The improvement in back pain was detected using subjective scoring with a VAS scale during the visits, which corroborated the results observed by the weekly VAS assessment performed at home. Significant improvements vs. placebo was detected as early as during the first week of intake Although a slightly better analgesic efficacy was observed in the high dose group, no significant differences were observed during any visit when comparing the two experimental doses. This finding suggests that the product effectively reduces pain at both doses used. In addition, the reduced pain sensitivity resulted in a significant reduction in analgesic medication among subjects taking the botanical blend, compared to the placebo group. This effect increased consistently over the entire study period.

The analgesic effect of the investigational product is consistent with the pain relieving properties described for the individual components. In relation to *Rosmarinus officinalis* L., different reviews have highlighted various medicinal properties, including antitumoral, anti-inflammatory, analgesic, neurodegenerative, endocrinal, antiinfective and antioxidant effects (28, 29). *R. officinalis* is mainly composed of polyphenols (such as apigenin, diosmin, luteolin, and phenolic acids especially rosmarinic acid) and terpenes (such pirosmanol, carnosol, carnosic acid, ursolic acid and oleanolic acid), which account for the beneficial therapeutic applications and pleiotropic use of rosemary. It has been shown that polyphenols found in plant extracts have antinociceptive affects, with attenuation of neuropathic pain in animal models as well as nociceptive and inflammatory pain (30). Moreover, the dipertenoids carnosol and carnosic acid exert anti-inflammatory and analgesic activities, interfering with the multiple signaling pathways that are deregulated during inflammation and underlying mechanisms of nociceptive pain (31).

Other components of the investigational product were ashwagandha root and sesame seed. Studies have shown that ashwagandha can help relieve persistent pain. For example, a randomized double-blind placebo-controlled trial using a standardized aqueous extract of roots plus leaves of *W. somnifera* (125 and 250 mg) administered for 12 weeks in patients with knee joint pain and discomfort, reported a significant pain reduction at 4 weeks compared to placebo (32). Many other benefits in a wide range of conditions related to immunomodulatory, cardioprotective, neuroprotective, antiaging, anti-stress/adaptogenic, anti-cancer, and anti-diabetic pharmacological activities of this phytochemical have been reported (33). On the other hand, sesamin, an active compound present in *Sesamum indicum*, has been shown to attenuate joint pain in osteoarthritis (34, 35).

Besides improvements in pain intensity according to VAS scores, objective measurements based on the PROMIS-29 and Cornell questionnaires showed overall better results in subjects treated with the investigational product. In the PROMIS-29, pain intensity decreased to a greater extent in both groups (low and high dose) of the investigational product compared to placebo. Although the reduction was slightly greater in the group that took the highest dose, no significant differences were observed in any of the visits when comparing the two doses. In the domain of physical function improvements, the results were also higher in the investigational product groups com-pared to the placebo group at the end of the study. This result was strengthened by the fact that clinically relevant changes in the Pain Disability Roland Morris Questionnaire were found in the groups treated with the investigational ingredient.

In the Cornell questionnaire, a significant improvement in pain relief in the cervical and upper back areas was observed after treatment with the investigational product compared to placebo, in particular in the high dose group. However, due to the limited number of subjects with shoulder pain, the results were not statistically significant, especially in the group that received a low dose of the investigational product. Thus, further studies are required with a larger sample size of individuals suffering from musculoskeletal shoulder pain to draw definitive conclusions.

Pain reduction might be accompanied by an improvement in QoL. In general, subjects assigned to the investigational product, especially those in the high dose group, showed significantly greater improvements from baseline to the end of the study in the domains of the SF-36 questionnaire of bodily function and health transition than those treated with placebo. There are numerous studies demonstrating that herb/botanical supplements may improve the quality of life in patients with different conditions (36). Previous studies have established connections between pain perception and emotional states (37). In addition, numerous studies have shown that patients with chronic musculoskeletal pain including lower back pain, osteoarthritis, rheumatoid arthritis, and fibromyalgia, tend to have a higher incidence of depression, anxiety, and sleep disturbance (38–40). This highlights the importance of a comprehensive approach to pain management.

The results of our study showed that although the investigational product did not exhibit superiority over the placebo in reducing depression symptoms, anxiety level and stress across the entire study population, a subgroup analysis revealed promising outcome. In a secondary analysis selecting subjects with minimal symptoms (those with scores of ≥4 for BDI-II, ≥ 14 for STAI-state, and ≥ 16 for PSS) subjects treated with the investigational product showed significant improvements in the levels of depression, anxiety, and perceived stress, which was particularly noticeable in the high dose group. Therefore, improvements seen after consumption of the investigational product in subjects with minor symptoms further support the beneficial effect of the product, especially at high doses. This aligns with previous studies emphasizing the bidirectional relationship between pain and emotional wellbeing. In addition to the reduction of pain facilitated by the investigational product, the improvements in emotional wellbeing observed in our study could be attributed to the presence in the investigational product of *Withania somnifera*, a plant with recognized adaptogenic properties. *Withania somnifera* has been used in traditional medicine to treat a variety of conditions, including anxiety and stress-related disorders (41).

On the other hand, the subgroup-specific improvements in depression, anxiety and stress levels highlight the importance of considering baseline conditions when assessing the efficacy of the investigational product and future studies should delve deeper into cohorts characterized by more pronounced emotional distress.

Additionally, there is evidence that pain and sleep quality are interconnected (42), and it is known that back pain increases the presence of sleep disturbances, which may trigger disability and depressive symptoms. At the end of the study, the high dose of the investigational product resulted in an improvement in the quality of sleep evaluated by the PSQI compared to the lower dose and the placebo groups. This was confirmed in the sleep quality assessment by actigraphy, particularly in the sleep latency domain. Herbal medicinal products are widely considered natural alternatives to common medication to help with sleep disorders and treatment of insomnia (43, 44). However, poor methodology of many studies restricts any clear conclusion. The present findings should be interpreted taking into account some limitations of the study, such as the reduced sample size and the fact that the dietary supplement was not administered beyond 12 weeks. However, the validity of the present findings is supported by the design of the study as a randomized double-blind placebo controlled trial and the number of different variables that have been evaluated using validated instruments to achieve the study objectives. Moreover, two daily doses (400 and 800 mg) of the botanical extract supplement were evaluated to determine whether there were significant differences in efficacy. Two studies included in the systematic review of Oltean et al. (15) evaluated low and high doses of herbal products in patients with exacerbated episodes of back pain (45, 46). In a randomized double-blind controlled study of 197 subjects with exacerbations of chronic low back pain, an oral *Harpagophytum* extract containing 50 and 100 mg of the marker harpagoside was administered, and the benefits appeared to be greater than placebo in the two dose groups (*p* = 0.027) (45). In the second randomized controlled study of 210 patients with an exacerbation of back pain assigned to receive an oral willow bark extract with either 120 mg (low dose) or 240 mg (high dose) of salicin or placebo, the percentages of pain-free patients were 39% in the high-dose extract, 21% in the low-dose extract, and 6% in the placebo group (*p* < 0.001) (46). In the present study, the high dose of the investigational product appeared to have more pronounced effects than the lower dose, although without statistically significant differences, improvement of some domains of QoL, emotional wellbeing, and perceived sleep quality. Overall, the safety of the product and its effectiveness in reducing pain provide the basis for further studies of prolonged administration in a larger study population of subjects with musculoskeletal back pain.

## 5 Conclusion

Dietary supplementation for 12 weeks of a blend of polyphenolic standardized extracts of rosemary, ashwagandha, and sesame was effective in reducing the intensity of pain in subjects with chronic myofascial cervical and back pain. The effect of the investigational product was significantly higher compared to placebo at both dosages studied.

Furthermore, the positive effects of the investigational product on emotional wellbeing were demonstrated among subjects with mild manifestations, particularly with the high dose (800 mg daily). Beyond pain relief, the investigational product demonstrated benefits in enhancing the quality of life (QoL) and improving sleep quality among the study participants. The investigational product was safe and well tolerated, which justifies the design of future controlled clinical trials with a larger sample size and a more prolonged administration period.

## Data availability statement

The original contributions presented in the study are included in the article/Supplementary material, further inquiries can be directed to the corresponding author.

## Ethics statement

The studies involving humans were approved by Comité de Ética de la Investigación de la UCAM. The studies were conducted in accordance with the local legislation and institutional requirements. The participants provided their written informed consent to participate in this study.

## Author contributions

SP-P: Investigation, Methodology, Software, Supervision, Validation, Writing – original draft, Writing – review & editing. JM-C: Investigation, Software, Validation, Writing – original draft, Writing – review & editing. JT: Investigation, Validation, Writing – original draft, Writing – review & editing. AL-R: Investigation, Writing – review & editing. JM: Investigation, Writing – review & editing. MM-C: Investigation, Writing – original draft, Writing – review & editing. MD: Investigation, Writing – review & editing. EV: Investigation, Writing – review & editing. VÁ-G: Investigation, Writing – review & editing. NC: Visualization, Writing – review & editing. PN: Visualization, Writing – review & editing. MC: Visualization, Writing – review & editing. FJL-R: Data curation, Formal Analysis, Methodology, Validation, Writing – original draft, Writing – review & editing.
