# Supplementary material for: Dietary supplementation with plant extracts for amelioration of persistent myofascial discomfort in the cervical and back regions: a randomized double-blind controlled study
Source: Front Nutr. 2024 Jun 3;11:1403108. doi: 10.3389/fnut.2024.1403108 (PMC11182357; doi:10.3389/fnut.2024.1403108)
Supplement: Supplementary file 1 [file Table_1.DOCX]

Table S1. Changes in quality of life in the three study groups according to the WHOQOL-BREF

| WHOQOL-BREF  domains and  study groups | Mean ± SD scores | | | | Within- group  *p* value | Between- group  *p* value |
| --- | --- | --- | --- | --- | --- | --- |
|  | Visit 1  baseline | Visit 2  28 days | Visit 3  56 days | Visit 4 (final)  84 days |  |  |
| Overall quality of life |  |  |  |  |  |  |
| Placebo (n = 42) | 3.8 ± 0.6 | 3.7 ± 0.6 | 3.8 ± 0.5 | 3.8 ± 0.6 | 1.000 | 0.219 |
| Low dose (n = 39) | 3.5 ± 0.8 | 3.7 ± 0.9 | 3.7 ± 0.8 | 3.9 ± 0.8* | 0.001 |  |
| High dose (n = 42) | 3.7 ± 0.9 | 3.8 ± 0.9 | 3.8 ± 0.9 | 4.0 ± 0.9* | 0.026 |  |
| Overall general health |  |  |  |  |  |  |
| Placebo (n = 42) | 3.4 ± 0.8 | 3.4 ± 0.9 | 3.5 ± 0.7 | 3.5 ± 0.7 | 0.668 | 0.693 |
| Low dose (n = 39) | 3.3 ± 0.9 | 3.4 ± 0.9 | 3.5 ± 1.0 | 3.7 ± 0.8* | 0.003 |  |
| High dose (n = 42) | 3.5 ± 1.1 | 3.6 ± 1.0 | 3.6 ± 0.9 | 3.8 ± 0.9* | 0.050 |  |
| Physical health |  |  |  |  |  |  |
| Placebo (n = 42) | 26.1 ± 3.8 | 26.3 ± 3.9 | 26.8 ± 3.8 | 26.7 ± 3.6 | 1.000 | 0.209 |
| Low dose (n = 39) | 25.9 ± 4.7 | 27.2 ± 4.2 | 27.6 ± 4.0* | 27.6 ± 6.0* | 0.050 |  |
| High dose (n = 42) | 24.7 ± 5.3 | 26.2 ± 5.8* | 26.9 ± 5.1* | 27.6 ± 5.2* | 0.001 |  |
| Psychological |  |  |  |  |  |  |
| Placebo (n = 42) | 22.5 ± 3.1 | 22.6 ± 3.1 | 22.3 ± 3.1 | 22.5 ± 3.3 | 1.000 | 0.036 |
| Low dose (n = 39) | 22.3 ± 4.2 | 22.6 ± 3.5 | 22.7 ± 3.9 | 23.2 ± 3.6 | 0.203 |  |
| High dose (n = 42) | 21.7 ± 4.1 | 22.4 ± 4.2 | 22.8 ± 4.3* | 23.2 ± 4.5* | 0.001 |  |
| Social relationships |  |  |  |  |  |  |
| Placebo (n = 42) | 11.5 ± 2.5 | 11.3 ± 2.5 | 11.6 ± 2.3 | 11.6 ± 2.4 | 1.000 | 0.909 |
| Low dose (n = 39) | 11.7 ± 1.9 | 11.5 ± 1.9 | 11.6 ± 2.3 | 11.6 ± 1.9 | 1.000 |  |
| High dose (n = 42) | 11.6 ± 2.7 | 11.8 ± 2.7 | 11.9 ± 2.4 | 12.0 ± 2.6 | 1.000 |  |
| Environment |  |  |  |  |  |  |
| Placebo (n = 42) | 29.0 ± 4.8 | 29.0 ± 4.8 | 29.1 ± 5.0 | 29.4 ± 4.7 | 1.000 | 0.934 |
| Low dose (n = 39) | 29.4 ± 5.2 | 29.3 ± 5.0 | 29.7 ± 5.3 | 29.8 ± 4.7 | 1.000 |  |
| High dose (n = 42) | 29.2 ± 4.6 | 29.1 ± 5.3 | 29.9 ± 5.5 | 30.1 ± 5.4 | 0.488 |  |

SD: standard deviation; asterisks indicate statistical significance.
