# Supplementary material for: Dietary supplementation with plant extracts for amelioration of persistent myofascial discomfort in the cervical and back regions: a randomized double-blind controlled study
Source: Front Nutr. 2024 Jun 3;11:1403108. doi: 10.3389/fnut.2024.1403108 (PMC11182357; doi:10.3389/fnut.2024.1403108)
Supplement: Supplementary file 2 [file Table_2.DOCX]

Table S2. Changes in serum biochemical markers at the end of the study as compared with baseline in the three study groups

| Biochemical marker | Baseline | End of study | *P* value |
| --- | --- | --- | --- |
| C-reactive protein, mg/dL |  |  |  |
| Placebo (n = 42) | 0.36 ± 0.48 | 0.17 ± 0.22 | 0.632 |
| Low dose (n = 39) | 0.47 ± 0.50 | 0.19 ± 0.26 |  |
| High dose (n = 42) | 0.33 ± 0.50 | 0.14 ± 0.24 |  |
| IL-6, pg/dL |  |  |  |
| Placebo (n = 42) | 2.06 ± 1.13 | 2.06 ± 1.28 | 0.978 |
| Low dose (n = 39) | 2.05 ± 0.87 | 2.03 ± 0.94 |  |
| High dose (n = 42) | 2.10 ± 1.19 | 2.06 ± 1.08 |  |

Data as mean ± standard deviation.
